# Embracing a different outlook: Strengths and goals of individuals currently in treatment for anorexia nervosa

**DOI:** 10.1007/s40519-024-01689-x

**Published:** 2024-10-02

**Authors:** Kelly M. Dann, Amy Harrison, Aaron Veldre, Phillipa Hay, Stephen Touyz

**Affiliations:** 1 School of Psychology, The University of Sydney, Sydney, Australia; 2 Faculty of Medicine and Health, InsideOut Institute for Eating Disorders, The University of Sydney and Sydney Local Area Health District, Charles Perkins Centre, Sydney, NSW 2006 Australia; 3 Department of Psychology and Human Development, University College London, London, UK; 4 Graduate School of Health, University of Technology Sydney, Sydney, Australia; 5 School of Medicine, Translational Health Research Institute, Western Sydney University, Sydney, Australia; 6 Mental Health Services, SWSLHD, Camden and Campbelltown Hospitals, Liverpool, NSW Australia

**Keywords:** Anorexia nervosa, Eating disorders, Recovery, Strengths, Goals, Positive psychology, Personal recovery

## Abstract

**Purpose:**

Developing personal goals beyond weight and shape, and promoting the agency to pursue those goals, could aid in treatment and recovery from anorexia nervosa (AN). This research explores the strengths, interests and goals of individuals currently receiving treatment for AN and evaluates how treatment services are supporting them to work towards personal goals across all areas of everyday life.

**Method:**

A total of 58 community-dwelling adults currently receiving treatment for anorexia nervosa at any stage of recovery completed the Client Assessment of Strengths, Interests and Goals Self-Report (CASIG-SR). Participants reported their goals for accommodation, work and study, interpersonal relationships, recreational activities, spirituality, religion or life purpose, physical health and mental health, and the personal strengths and supports needed to achieve those goals. Concordance scores were calculated between importance of personal goals and level of support from current services regarding these goals.

**Results:**

Themes identified across goals, strengths and supports were *Connection*, *Independence & Confidence, Meaning & Self: The Real Me, and Stability & Balance*. Work and study goals and strengths were identified strongly. The key support needed was stability from the current treatment team to provide a stable base for change. Concordance scores indicate support provided for personal goals was less than the importance of the goal to the individual.

**Conclusion:**

Results suggest goals for everyday living are critical to recovery in anorexia nervosa. Specific clinical considerations to increase motivation and hope are increased access to peer support, a focus on increasing positive affect, supporting safe exercise and promoting outdoor experiences and connection with nature.

**Level III:**

Evidence obtained from well-designed cohort or case–control analytic studies.

## Background

Anorexia nervosa (AN) is a psychological disorder which causes large and sometimes lengthy disruptions to individuals’ lives. Medical complications may necessitate hospitalisation, and can involve an extended duration of treatment, and considerable impact on normal functioning across all areas of everyday life [1–4]. Maintaining hope can be difficult during a long course of illness. But hope − needed to sustain motivation and belief in the possibility of change − is a key factor in recovery from AN [5]. It has been over 20 years since Irving and Cannon (2002) in *Starving for Hope* described how goals and goal-pursuit behaviours may operate not only in the development and maintenance of eating disorders but also in their treatment and recovery.

‘*Recovery necessitates learning how to cope with stressors and challenges using healthy means and discovering things other than weight and appearance that bring joy and pleasure, that provide a sense of control, purpose and meaning, and that can anchor and guide future actions’* [6, p 280].

Recovery from an eating disorder is strongly associated with re-engagement with work, study, leisure activities and interpersonal relationships, and improvements in everyday functioning are often rated as the most important outcomes of treatment [7]. How to best incorporate these areas of personal recovery with medical recovery has not reached consensus [8–10]. However, clinical guidelines for AN include principles for recovery-oriented practice [11] such as recognizing personal strengths and maximizing self-determination, which are consistent with the concept of recovery as a personal process which prioritizes hope, autonomy, connectedness and the development of meaning and purpose [12]. Evidence-based treatments for eating disorders in adults generally include some recognition of the importance of personal strengths, but most do not explicitly adopt a strengths-based approach to care. When strengths exercises are included in therapy, they appear to be well received by patients with eating disorders [13, 14], and have been piloted in inpatient treatment settings with positive results [15, 16]. Understandably, the severity of AN requires a clinical focus on the reduction of core disorder-related cognitions and behaviours, and the restoration and maintenance of BMI. However, the focus on food, weight and shape in treatment may be problematic, as it mirrors the unhelpful focus on food, weight and shape that it is trying to change [7]. Despite current illness, individuals with AN are noted to maintain high engagement in work and study and demonstrate areas of strength in their everyday functioning [17]. Incorporating a specific focus on strengths and goals for everyday living into treatment could balance the attention to reduction of disorder-related thoughts and behaviours, and ‘build-what’s-strong’ not just ‘fix-what’s-wrong’ [18, p 631].

### Strengths assessments

A strengths-based approach begins with an assessment of needs and strengths, of the individual, of their environment and at the interpersonal level where they interact [19]. Strength assessments are useful at the beginning of treatment, as matching the goals of therapy to the most important goals of the individual has been demonstrated to increase motivation and result in greater satisfaction when they are achieved. However, even the process of completing a strengths assessment without it being explicitly tied to treatment can result in positive outcomes, such as improvements in mood and increased likelihood of personal goal setting (see Bird et. al., 2012 for a review).

The Client Assessment of Strengths, Interests and Goals Self-Report [CASIG-SR; 20] is a psychometrically validated measure developed to assess functional outcomes in individuals with psychiatric disorders. In a clinical setting, where disorder-specific symptoms are necessarily a focus of treatment, the importance of everyday functioning − especially work, study and independent living − may be underestimated [21]. The CASIG-SR is designed to identify areas of need between current functioning and personal goals, so that support services can target the areas of need that are most important to the client. Research with individuals living with a serious psychiatric disorder utilized the CASIG-SR to identify personal goals, but also asked participants to rate 1. the importance of working towards the goal, and 2. the extent to which their current support services were helping them work towards the goal [22]. Ratings indicated that goals for everyday living such as work, study and improving physical health and interpersonal relationship were most important to respondents, but concordance between the ratings indicated that support services were more helpful with mental health-specific goals [22]. The CASIG-SR has been assessed as suitable for routine use in healthcare settings [19]. Considering the strong association between improvements in everyday functioning and recovery in individuals with eating disorders, the CASIG-SR may be a useful clinical measure in AN, but its use has not been explored in this population.

### Aim

The aim of the current research was to explore the use of the CASIG-SR in individuals currently receiving treatment for AN, and to better understand their strengths, interests and goals and how treatment services are supporting them to work towards personal goals in all areas of their lives.

## Method

### Participants and procedure

Participants were 58 individuals currently receiving treatment for AN recruited from the community. Participants were eligible if they were 18 years old or over, had a current formal diagnosis of AN (including atypical AN) from a health professional and were currently receiving treatment or support for that diagnosis. Participants at any stage of recovery were included. This study was advertised on university and eating disorder organization websites and their related newsletters and social media channels. Data were collected via an online survey and participants provided informed consent before beginning this study, which was approved by The University of Sydney Human Research Ethics Committee (2021/838).

### Measures

***The Client Assessment of Strengths, Interests and Goals Self-Report ***(CASIG-SR; 20) assessed personal goals for accommodation, work and study, interpersonal relationships, recreational activities, spirituality, religion or life purpose, physical health and mental health. For each life area, participants were asked if they had a goal for the upcoming year (Yes/ No). For each goal, they were asked a series of open-text response questions: ‘What are your (work or education) goals?’ ‘What strengths do you currently have to help you achieve these goals?’ and ‘What kind of help would you need to help you achieve these goals?’ Following Lecomte et al. [22], two additional questions were included: ‘To what extent is working towards these goals important to you at this time?’ rated on a Likert scale (1: Not at all important—4: Very important), and ‘To what extent do you consider the services that you are currently receiving are helping you to work towards achieving these goals?’ rated on a Likert scale (1: Not helpful—4: Very helpful). Ratings for these questions were used to calculate a concordance score which indicated the level to which the services are meeting the needs of the individual regarding their goals. Participants were provided with an open-text field for optional additional feedback at the end of the survey.

### Data analysis

Open-ended response data were analysed using interpretive content analysis [23], a method which allows the combination of qualitative and quantitative description of the data. Constant comparative analysis was employed to open-code both manifest (visible, literal) and latent (underlying, inferred) meaning in the text [23]. Text extracts were compared to identify and code those with similar concepts, and code groups were then compared to identify patterns and create higher-level categories or themes. To improve the validity of coding of latent content and category grouping, both authors KD and AV reviewed the material. Manifest language from the data extracts was used to inform the naming of conceptual codes and categories. When all codes and categories were finalized, the entire data set was re-examined to reduce the risk of data omission. Exemplar quotes were re-examined by participant ID to ensure a broad representation across participants.

Goals, strengths and supports were all reported within the context of life areas. However, goals were often specific to the area, while strengths and supports were often common across more than one area. Therefore, to best represent the content, the most frequent goals are presented by life area as subthemes. This allows comparison across life areas and prevents over-generalization of the content, which is particularly important as goals should be specific. To best represent the number of strengths generated, and their breadth across individual, environmental and interpersonal domains, the most frequent strengths mentioned are reported across all life areas. Supports needed are also reported across life areas. The main themes reported comprise content from goals, strengths and supports, and general feedback.

Concordance scores for the treatment question were calculated from rating data using the equation: *1—(importance of goal—help from service) / importance of goal*. A score of 1 indicates support from services matches the importance of the goal, scores less than 1 indicate the service does not meet the importance of the goal and scores greater than 1 indicate the service exceeds the importance of the goal. Quantitative analysis was conducted using SPSS 28 [24] and NVivo 20 was used to manage qualitative data coding.

## Results

Participants were mostly women (no men responded to the survey) aged between 18 and 51 years. Most participants had tertiary education, and nearly all were working or studying. Most participants had multiple support services, with a wide range of years of support (6 months to 30 years). Full participant demographics are reported in Table 1.

**Table 1:** Sample demographic and clinical characteristics

Characteristic	*N*	*M* (*SD*) or %
Age (years)	58	28.7 (7.8)
Gender		
Female	55	95%
Non-binary	3	5%
Ethnicity		
Caucasian	53	91.4%
Other	5	8.6%
Highest level of education		
University postgraduate	13	22.4%
University undergraduate	29	50%
Tertiary certificate or diploma	7	12.1%
Higher School Certificate (Yr. 12)	7	12.1%
School Certificate (Yr. 10)	2	3.4%
Occupation		
Working Full-Time	15	25.9%
Working Part-Time	16	27.6%
Not Working/ Volunteer	6	8.6%
Studying	8	13.8%
Studying and Working	13	22.4%
AN Diagnostic subtype		
AN-R	39	67.2%
AN-BP	9	15.5%
OSFED/ Atypical AN	9	15.6%
EDNOS-AN	1	1.7%
Total years of support	58	7.1 (7)
Current support service providers		
General Practitioner	50	86.2%
Psychologist	45	77.5%
Dietician	33	56.9%
Psychiatrist	30	51.7%
Allied Health	5	8.6%
Recovery Coach	4	6.9%
Other therapist (Psychotherapy, Art)	3	5.2%

Most people (*N* = 50, 86%) were receiving more than one form of support; therefore, support providers do not total to 100%

The most frequently mentioned goals and their concordance scores are presented by life area in Table 2.

**Table 2:** Sub-themes identified in goals and concordance scores by life area

Goals	Exemplar	*N*	Concordance
Accommodation		22	0.82
Independent living	*‘I would like to live in my own house’*		
Connection to nature	*‘I would love to be able to be in nature and around animals’*		
Work and education		40	0.77
Growth and change	*‘Would love to do post-graduate study and upskill in the workplace’*		
Meaning and ‘giving back’	*‘I want to help people feel listened to, and have their options and treatments explained, questions answered, and help people access the services they need’*		
Interpersonal relationships		47	0.80
More connections	*‘I want to be able to see friends and loved-ones more, reply to messages sooner, maintain relationships, socialise more’*		
True connections	*‘I want to build better friendships’*		
The real me	*‘Isolate less and be able to engage with others as my true self’*		
Recreational activities		38	0.81
Activities for joy and more relaxing hobbies	*‘My goal is to be able to do activities for joy and not out of compulsion’* *‘Do more calming activities like reading’*		
Back to past activities	*‘I’d love to be able to dance again’*		
Physical health		43	0.86
Energy and strength	*‘My goal is to feel healthy—which for me starts with getting my energy back!’*		
Reduce pain and other symptoms	*‘Less pain and less other troubling symptoms’*		
Mental health		49	0.81
Improve mood	*‘I want to be able to feel happiness again’*		
Eating disorder recovery	*‘I want to become free from my eating disorder, move on with life’*		
Self-compassion	*‘allow myself rest and kindness’*		
Spirituality, religion or life purpose		17	0.76
Religion	*‘become an active member of my church family’*		
Spirituality, peace and self-care	*‘I want to find a greater sense of peace within myself’*		

The most frequently mentioned strengths across all life areas are reported in Table 3. The most frequently mentioned supports needed are presented in Table 4.

**Table 3:** Most frequently mentioned strengths across all life areas

Strengths	Exemplar	*N*
Personal qualities		
Dedicated, determined	*‘My determination is a strength’*	15
Perseverant, consistent	*‘Stubbornness to not let my ED control me’*	15
Love learning, quick learner	*‘My strengths include a love of learning’*	14
Organized	*‘high level of organization skills’*	10
Kind, caring	*‘I am a caring and kind person’*	9
Good communication skills	*‘can connect with people easily’*	8
Creative	*‘I am creative’*	7
Time management, planning	*‘I am pretty organised and like to plan ahead’*	7
Strong work ethic	*‘I am a hard-working person’*	6
Resilient	*‘Resilience and I have good support network’*	6
Social, friendly	*‘I seem to put people at ease’*	5
Good listener, non-judgmental	*‘I am great at listening to people, and not judging them’*	5
Empathetic	*‘I feel like I am empathetic’*	5
Patient	*‘more patience and tolerance than I used to have for goals that take time’*	4
Resourceful, problem-solver	*‘problem solving skills to plan ahead as to what may come up and what I may need’*	4
Brave, adventurous	*‘I am brave at taking on new challenges’*	4
Intelligent	*‘Good education, intelligence’*	4
Reliable	*'I am reliable’*	3
Experience		
Life experience	*‘Insight based on past experience’*	31
Self-understanding	*‘I know what I value and what I like’*	23
Education	*‘I have an undergraduate degree’*	22
Therapy skills	*‘I have skills and resources from previous treatment’*	21
Recovery progress	*‘recovery has made me realise I can do difficult things’*	7
Lifetime memories	*‘I used to like to try new things’*	5
Independent living skills		
Work experience	*‘I have worked in many different areas’*	16
Self-care skills	*‘Able to manage self independently’*	8
Current job	*‘I earn enough to live by myself’*	6
Independent living skills n/s	*‘Independent living skills, and financial stability’*	6
Interpersonal		
Treatment team	*‘I have a supportive treatment team’*	16
Friends	*‘I have good friends’*	9
Family	*‘I have support from my family’*	6
Partner	*‘my partner is very patient’*	6
Support network n/s	*‘lots of support’*	7
Doubts		
No strengths	*‘None’*	29

**Table 4:** Most frequently mentioned supports needed

Supports needed	Exemplar	*N*
Ongoing eating disorder & mental health support, ‘current treatment team’	*‘Current team (psychologist, dietitian, GP) to keep my eating disorder recovery on track’*	98
Extra support	*‘health professionals that are neurodiversity-affirming and inclusive’*	35
Family and friends	*‘I need support from family and friends during challenging times’*	32
Eating disorder support not specified	*‘I need support with recovery from anorexia’*	20
Stress, self-confidence, mood	*‘More self-confidence support’*	13
Work skills and planning	*‘resume, cover-letter and interview skills’*	9
Peer-support, mentoring ED	*‘peer support groups for ED mental health recovery would be really helpful’*	6
Workplace support/mentoring	*‘mentorship and supervision from superiors’*	5
Doubts	*‘I’m not sure, nobody can seem to help’*	5

### Main themes across goals, strengths and supports

#### ***Connection***

Across all life areas, the strongest theme was connection − connection with the community, with nature, with family, friends and partners, and connection with the true self. Connection was the most frequently mentioned goal for interpersonal relationships − to improve both the quantity and quality of relationships, and to build stronger relationships, and to ‘be present’ when connecting with others.

‘*I want to be able to see friends and loved ones more, reply to messages sooner, maintain relationships, socialise more’*. *(Interpersonal relationship goal)*

*‘I would like to be more present in my interpersonal relationships. I would like to be able to engage on a deeper level, listen and actually hear what they are saying’. (Interpersonal relationship goal)*

Participants noted personal and interpersonal strengths related to connection; however, goals for interpersonal connections were also paired with doubts about the strengths needed to attain the goals. Across all life areas, participants reported the greatest number of doubts about their current strengths in reference to their interpersonal relationship goals.

Making changes to increase connection with the broader community was mentioned in spirituality, religion or life purpose goals, and accommodation goals*;* however, the key subtheme for accommodation goals was connection to nature. Participants mentioned goals for moving home to be closer to the mountains, to the sea, or more generally to be ‘in nature’, e.g. ‘*Remote temperate climate with lots of mountains. Somewhere very spacious and quiet’ (Accommodation goal).*

#### ***Independence ****&**** Readiness for change***

The most frequently expressed accommodation goal was of wanting to live independently, whether that be solo, or with a partner, children or pets. Independent living goals were strongly related to independent living skill strengths. Participants reported work and financial skills to support independent living, such as having current work, having education and skills to support finding work, having a strong work ethic and being good at saving money. Independent living goals were also supported by personal strengths such as self-reliance and being organized.

Across all life areas, work and education goals showed the most confidence, independence and readiness for change. Participants mentioned goals for upskilling or moving to the next level in the workplace or in their education, and related strengths in personal qualities such as communication skills, work ethic, determination, creativity and education-related strengths such as intelligence, having a love of learning and being a fast learner. Independence was noted in work and education goals to change to self-employment, less stressful work and more creative work. Work and study goals were strongly associated with motivation to change, and motivation to access support for those changes.

*‘Love to study, passionate about my career’ (Work and study strength)*

*‘to manage my anorexia better so I can get back to working full-time’ (Supports needed for work/study goals)*

#### *Meaning & Self:* *The Real Me *

The Real Me was a strong theme in interpersonal relationships − participants mentioned goals to be more open, to stop hiding, to express their feelings and to feel more comfortable around friends, family and partners.

*‘Being more open and comfortable around my mum. Not hiding from friends and family’ (Interpersonal relationship goal)*

Strengths associated with The Real Me were reported in relation to interpersonal relationship goals, e.g. *‘don’t want to pretend like being someone else’ (Interpersonal relationship strength),* but doubts about strengths were also related to The Real Me, e.g. ‘*it feels like new territory, relating to friends in a genuine way’ (Interpersonal relationship strength).*

Work and study goals were also strongly related to meaning and self, with many participants noting goals for finding occupations that held personal meaning. For many respondents, the goal was to use their experience to give back to the community. Participants mentioned work and study goals in aged care, allied health, medicine, nursing, peer work, psychiatry, psychology, school counselling, social work and teaching. Participants noted strengths related to past experiences that could support their goal to give back in meaningful community work.

*‘I want to start working at something I love, and have the courage to pursue that’ (Work and study goal)*

*‘Ability to see past a person’s mental illness and see the person stuck behind it’ (Work and study strength)*

Mental health goals and strengths were related to meaning and self-understanding, and particularly to self-compassion.

*'I want to feel I deserve to take up space in all ways’ (Mental health goal).*

Physical health goals for regaining energy and strength were also related to what that improvement could allow in personally meaningful areas:

*‘I would like to recover enough to be able to fall pregnant and have a healthy child’ (Physical health goal*)

Goals for recreational activities also related to getting back to personally meaningful past activities, to get back to The Real Me, e.g. ‘*I’d love to be able to dance again’, ‘get back into surfing’ (Recreation goals).*

#### ***Stability & Balance***

Stability of care was the most frequently mentioned support needed for goals across all life areas. The consistency of ongoing support from the current treatment team was noted as critical to goal attainment, to provide a stable base for change. The most frequently mentioned mental health goal was to improve and balance mood, and mood and eating disorder symptoms were often mentioned together:

*‘I want to feel less stuck in a cycle of anxiety /depression /ED behaviours’ (Mental health goal)*

Mental health goals were strongly related to stability in relation to recovery, and to balance in relation to decreasing the impact of disorder-related thoughts on life:

*‘I want to become free from my eating disorder, move on with life’ (Mental health goal)*

Goals for mood stability were also expressed as a more general increase in positivity—to have a ‘*more positive mindset’*, or to ‘*look forward to waking up’ (Mental health goals).* Strengths themselves were explicitly mentioned in terms of balancing change and acceptance:

‘*Embracing a different outlook … learning to accept my strengths and limitations’ (Mental health goal).*

Spirituality, religion or life purpose goals were related to seeking the stability and balance of inner peace, through spiritual practice, mindful activities such as meditation and yoga, and re-balancing priorities. Balance was also a strong theme in recreational activities, with many participants reporting goals related to re-balancing their approach to activity, specifically to increase engagement in activities for joy and to try more relaxing hobbies.

*'developing some hobbies that aren’t exercising’ (Recreation goal)*

Physical health goals were related to regaining stable and balanced bodily functioning. Participants mentioned wanting to improve their physical health to reduce physical pain, to normalize their gastro-intestinal function, to improve the health of their bones, skin, and hair and to resume menstruation.

##### Concordance scores

Concordance between the current importance of working towards personal goals, and the extent to which current services are helping to work towards those goal was relatively stable across life areas and ranged between 0.76 and 0.86 (see Table 2). This indicates that although services were providing support for current life goals, the level of support was less than the importance of the goal to the individual.

##### General feedback

##### ***Goals are critical to recovery***

Eight participants left further feedback about the role of goals, interests and strengths in their life, and their importance to ED recovery.

*‘I am glad you are looking at strengths and interests. One of the most helpful things for me in my recovery has been reconnecting with things that interested me before my anorexia took over my life' (General feedback)*

*‘People always focusing on you being unwell reinforces negative beliefs and creates a cycle. Putting the focus on other goals and strengths helps people to see that they are more than their eating disorder’ (General feedback)*

*‘These goals and relationships are what allow me to maintain my current level of health and ignore the feelings and thoughts that tell me to move back to a less healthy place. Current treatments for AN focus solely on the medical aspects and do not encourage these things, which is absolutely ridiculous given that the only way to beat it is to have a reason to’ (General feedback)*

## Discussion

This research explored the strengths and goals of community-dwelling adults currently receiving treatment for AN, and assessed to what extent support services are helping them to achieve their goals. Overall, goals were specific and well described, and participants generated a large number of strengths, across individual, environmental and interpersonal domains. The quality and detail of responses suggest that participants appreciated the chance to reflect on their goals and strengths, and to explore ‘possible selves’ [25], or visions of the future, in the present. Responses were generally written in the present or future tense, suggesting the survey promoted future-focused thinking. Reflecting on strengths and setting goals is also a useful way to link the past and the future, and responses included specific reference to this kind of looking-back-to-look-forward, e.g. *‘I have been in a good place before, so I know it’s possible’ (Spirituality strength).* Participant ratings indicated that their current support services are helping them work towards their goals, but that they wanted more − and more specific − support for their goals for everyday living, because they are critical to their recovery.

Across life areas, work and study goals and strengths were identified particularly strongly. Participants mentioned the importance of work and study to their life, and their responses are consistent with research which suggests reducing self-perception of disability and increasing employment is a critical factor in improving life satisfaction for individuals with AN [26]. Demographic information for the sample also shows high engagement in work and study despite current illness. Responses suggest work is associated with life satisfaction and independence; however, some respondents also mentioned work and study stressors are associated with eating disorder symptoms. Work and study therefore appear to play a functional role in the bidirectional relationship between quality of life and eating disorder symptoms [7]. The current results also provide strong support for the perspective that temperamental traits primarily viewed as risk factors for eating disorders (e.g. 27), are not negative per se, but rather adaptive or maladaptive depending on their focus [28]. Overall, the importance of work to quality of life demonstrated in the current study suggests individuals may need specific support for balanced workplace engagement and reinforces calls for systemic change to create safer work environments with which to engage [29].

Stability of support by current health services was the key theme for the supports needed for participants to work towards their goals. Best practice guidelines for AN indicate multidisciplinary support is needed, and most individuals in the current study had more than one type of support service, most commonly, a psychologist, a general practitioner and a dietitian. Access to ongoing support from the current treatment team was critical to the perceived feasibility of goal attainment, consistent with a meta-synthesis of treatment experiences in AN which found a strong therapeutic relationship supported readiness to change [30]. However, alongside the importance of stability, there was a strong wish for diversity in treatment, for more support with mood regulation and general well-being and for treatment to include support for everyday activities. Results of the current study are consistent with calls for an increase in the range of treatments available [31, 32]. The overall themes of goals and strengths in the current study align closely with the CHIME (connectedness, hope, identity, meaning and empowerment) framework of personal recovery in identified in mental health generally [19, 33], and eating disorders specifically [34].

The current results have important implications for all those supporting individuals with AN − both health professionals supporting people in their clinic, and families, carers and supports in the home environment. Firstly, the CASIG-SR appears to be well accepted by participants and may provide a useful clinical measure to explore interests and goals in this population. More broadly, the perspectives shared here allow a better understanding of the breadth of strengths, and the enduring importance of everyday goals, for those in treatment for AN. Finally, as lived experience perspectives on the importance of everyday strengths and goals during AN, this research has implications at a societal level, to ‘naturalize the state of living with an eating disorder’ [35]. Key goals for participants in this study were to be more connected to, and accepted by family, friends, co-workers and the community, to *‘be able to engage with life “normally” despite what may be going on with my mental health’ (Mental health goal),* to be understood and accepted for who they are *during* the eating disorder, not have validation tied to symptom remission. At a societal level, increased recognition of these strengths and goals may help de-stigmatize living with an eating disorder, and promote inclusivity [35, 36]. Themes identified in the current study also suggest specific clinical considerations, such as the utility of incorporating strengths-based approaches to improve the therapeutic alliance and incorporating more diverse treatments.

### Clinical considerations

#### ***Validation, motivation and hope***

Responses in the current study suggest a focus on goals and strengths promoted motivation for recovery, consistent with previous research which suggests when support providers recognize their strengths, individual with eating disorders feel validated, empowered and more capable of change [37]. A criticism of strengths-based approaches is that they focus solely on strengths and disregard (or make light of) weaknesses, in a way that may not be appropriate for clinical populations. However, exploring strengths in the context of goals, and considering the supports needed to work towards goals promotes reflection on relative strengths and weakness in a way that may be useful for treatment. Responses in the current study suggest participants were able to reflect on doubts about their strengths, and in some cases, to reframe their beliefs in a way that could sustain hope despite the ebb and flow, or the ‘rhythms of motivation’, observed clinically [38].

Individuals with AN often have an extended period of treatment, and over this time ‘carers hold the hope for recovery for our loved ones, until they can do it themselves, and we expect clinicians to do this too’ [31, p. 150]. Ongoing support from the current treatment team was a key theme, meaning clinicians have a great responsibility to provide stability of care, and ‘hold the hope’, possibly over a lengthy period. Can strengths-based approaches also help clinicians? Research in community mental health service providers has found greater use of recovery-oriented practices is associated with lower workplace turn-over, suggesting that it may boost job satisfaction and protect against clinician burnout [39]. And, although strengths-based assessments are primarily used between the patient and the clinician that administers the measure, with the patient’s permission, key goals and strengths can be an important addition to case consultation between clinicians so the wider team can help the individual to work towards their goals [40]. Although integration of multidisciplinary care should remain a priority for patients with AN, this is not the current reality for all, and respondents in this study mentioned that feeling understood by their clinicians was undermined by feeling like the ‘go-between person’, responsible for updating members of their treatment team. In cases where communication between clinicians falls to the patient, care plans that include specific life goals may be helpful, to allow patients to contextualize those updates within a framework of how they are working towards their goals.

#### ***Treatment diversity and hope***

*‘Instead of describing patients as “not engaged”, we could also be asking whether what is provided is particularly engaging, or made safe and hopeful enough to engage with’* [31, p. 151].

Ongoing support from the current treatment team was paired with a strong wish for additional and different supports. Other types of supports specifically mentioned were peer support, recovery coaches, physiotherapy, exercise physiologist, chiropractic, massage, naturopathic, physiotherapist-led yoga or Pilates groups, trauma counselling, exposure therapy, neurodiversity-affirming services, step-up to inpatient admission pathways, step-down outpatient services and specific support for food-related activities, friendships and intimacy issues. Participants also described their support needs in more general terms, as wishes for more creative and experiential therapies to engage with. Four key themes are discussed specifically in relation to wishes for treatment diversity, and the provision of engaging, safe and hopeful adjuncts to current care.

***Peer support, mentors and coaches.*** For goals across all areas of their lives, participants in the current study mentioned wanting support from peer supporters, mentors or coaches. The recovery model differs from the medical model in how expertise is defined—the recovery model values the expertise of personal experience and promotes peer support workers with lived experience [41]. For some individuals with AN, peer mentoring can be even more helpful than professional support [42]; it can help individuals feel understood, promote motivation for change and model resilience and hope [43]. Participants in the current research wanted to use the experience of living with AN to ‘give back’ to the community, to use their knowledge of the complexity of living with AN and navigating treatment to directly support others and to advocate for better care for individuals with AN. Peer support models are therefore also an opportunity for individuals in recovery to find positive ways to utilize their experiences.

***Exercise.*** Participants in the current study had goals for wanting to re-engage with safe forms of exercise and recognized that they would need support to do so. Exercise was specifically related to goals for increasing strength, reducing pain and being able to re-engage in everyday activities like gardening or walking the dog. Although unsupervised exercise is strictly limited in AN treatment, supervised exercise therapy intervention trials have found no adverse health outcomes and a range of benefits. Aerobic exercise is associated with cardiac and BMI improvement, resistance exercise is associated with muscle growth, and increased strength and mind–body exercise is associated with reduction in ED cognitions, anxiety and depression, and improvements in sleep and general quality of life [44, 45]. Guidelines for supervised re-introduction of exercise in eating disorder treatment have been proposed [46].

***Nature.*** Participants in the current study had specific goals to connect with nature, to increase their outdoor time and to engage in more relaxing leisure activities. Their wishes are consistent with preliminary evidence of the benefit of *friluftsliv*, or *free air life*, in eating disorder recovery [47]. Friluftsliv is a Norwegian term for the sense of freedom and spiritual well-being associated with spending time in nature. Rather than being about activity or challenge-based encounters with nature, friluftsliv is about just being in nature: sleeping outdoors, sitting around campfires and mindfully engaging with the natural environment. Participants with eating disorders in a pilot study reported friluftsliv helped them feel more connected and less lonely, helped them to cope with stress and explore new ways of dealing with difficulties, and provided a way to connect with childhood memories and rediscover a new sense of self [47].

***Positive affect.*** The most frequently mentioned mental health goal in the current study was improvement in mood. The majority of treatments in AN focus on reductions in negative affect, but trials in major depressive disorder have demonstrated strong results for interventions which directly focus on increasing positive emotions and cognitions [48]. Positive affect appears to be lower in individuals with AN, and higher levels of non-acceptance of positive emotions are associated with higher levels of anxiety and depression [49]. Positive affect can be enhanced by practicing up-regulation of affect, to ‘accentuate the positive’ by catching yourself when you are feeling good, and consciously reflecting on the good feelings. Strength assessments may be a useful way to identify what feels good, and to work towards accentuating the positive affect generated by those activities.

## Strengths and limits

Strengths of the current study include the use of a psychometrically validated strengths assessment which has not previously been utilised in this population. The sample was nearly all female; therefore, the themes discussed may not generalize to non-female populations. Information regarding current diagnosis and treatment for AN was self-reported, and the decision not to administer a standardized eating disorder diagnostic instrument in this research was consistent with the study aim to focus on everyday life rather than disorder-related cognitions or behaviours. Mean age and length of illness in the sample was relatively high, and therefore, the current results may be most relevant for individuals who have a more long-standing disorder. However, eating disorders often develop during adolescence and can have a considerable impact on normal social and vocational development in the period of emerging adulthood [50]. There are no specific interventions for individuals at this middle phase of AN in emerging adulthood [51], a critical point in development for identity exploration and formation, and research to explore the utility of adjunct interventions to promote individual goals and strengths in adolescents with AN are warranted.

## What is already known

Over twenty years ago, Irving and Cannon [6] suggested that developing personal goals beyond weight and shape, and promoting the agency to pursue those goals, could aid in the treatment of AN. Preliminary research to support further use of strengths-based approaches in eating disorder treatment has yielded promising results, but further research to evaluate these approaches as an ‘active ingredient’ of treatment is needed [52].

## What this study adds

This study explored the strengths, interests and goals of people currently in treatment for AN. Responses strongly suggest that goals for everyday living are a critical recovery factor for individuals with AN. How can these results be used? At the clinical and interpersonal level, health workers, families and friends can use this knowledge to be more alert to the strengths and goals of the people they are caring for, and once they are made more transparent, to promote the agency to pursue those goals.

## Data Availability

No datasets were generated or analysed during the current study.
